# Social Determinants of Health Impact Cervical Cancer Stage at Presentation among Women in Zambia

**DOI:** 10.21203/rs.3.rs-8522732/v1

**Published:** 2026-02-23

**Authors:** Graciela M. Nogueras Gonzalez, Mehmet Enes Inam, Susan Msadabwe, Kamaria L Lee, Bernadette Njala, Dyness Sakala, Mwando Chitula, Jane R Montealegre, Susan Peterson, Elizabeth Chiao, Lilie L. Lin

**Affiliations:** The University of Texas MD Anderson Cancer Center; The University of Texas MD Anderson Cancer Center; Cancer Diseases Hospital; The University of Texas MD Anderson Cancer Center; Cancer Diseases Hospital; Cancer Diseases Hospital; Cancer Diseases Hospital; The University of Texas MD Anderson Cancer Center; The University of Texas MD Anderson Cancer Center; The University of Texas MD Anderson Cancer Center; The University of Texas MD Anderson Cancer Center

**Keywords:** Social Determinants of Health, Neoplasm Staging, Uterine Cervical Neoplasms, Epidemiology, Cross-Sectional Studies, Health Services Research, Developing Countries

## Abstract

**Background:**

The impact of social determinants of health (SDOH) on cervical cancer stage in Zambia remains poorly understood and understudied.

**Methods:**

We administered the Accountable Health Communities Health-Related Social Needs Screening Tool, modified for Zambian culture, to newly diagnosed cervical cancer patients at the Cancer Diseases Hospital (CDH) in Lusaka, Zambia. The primary aim of the study was to determine the relationship between sociodemographic factors, SDOH domains, and advanced cervical cancer stage (III/IV) at presentation to CDH. Logistic regression models were performed to determine associations.

**Results:**

Between June 2022 and March 2025, there were 259 survey respondents. Their median age was 50 years (range: 29–78), 47.1% of them were diagnosed with advanced cervical cancer, 55.2% were women living with HIV, and most (92.6%) had completed primary or secondary education. Although none of the standard SDOH domains including education, housing, transportation, and rurality were associated with advanced cancer stage, lack of knowledge about cervical cancer risks and symptoms (OR = 1.79, 95% CI: 1.03–3.11; p = 0.04) was the main factor associated with advanced cervical cancer stage in the adjusted model.

**Conclusions:**

Awareness about risks and symptoms of cervical cancer was the only factor associated with advanced cancer stage at presentation. While additional studies are needed to understand how SDOH may contribute specifically to cervical cancer prevention and diagnostic services access in low- and middle-income countries, improving educational strategies to increase awareness of the importance of screening and early diagnosis could reduce late-stage cervical cancer presentations.

## Novelty and Impact

We observed a significant association between awareness of cervical cancer risks and symptoms and FIGO stage which provide new insights that could benefit further research and practical application as we determine the best interventional strategies to test in the future. We believe these findings advance the field by addressing critical epidemiologic gaps and offering evidence to guide targeted strategies for health education and screening programs.

## INTRODUCTION

Social determinants of health (SDOH) encompass non-medical factors that impact patient health, including marital status, educational level, neighborhood disadvantage, social support, income status, and patient health literacy.^1^ SDOH factors have been linked to obstacles in accessing timely care for cancer patients, resulting in advanced-stage diagnoses and poorer outcomes. Impacts of SDOH on cancer outcomes, including late stage of diagnosis have been documented in well-developed countries such as the U.K. and Australia.^2–4^

Cervical cancer is one of the most preventable and treatable cancers, but remains a significant global health burden.^5^ In low- and middle-income countries (LMICs) like Zambia, the impact of SDOH, including healthcare access barriers, including health literacy is even more pronounced.^6–8^ In Zambia as in other countries in Southern Africa, cervical cancer remains the leading cause of cancer incidence and mortality.^9^ Healthcare access barriers may contribute to lengthy delays in diagnosis and treatment, highlighting the urgent need for targeted research and policy interventions that enhance cancer care delivery in resource-limited settings.^10–12^

Research on SDOH and cervical cancer in LMICs like Zambia remains limited. This study aims to understand the relationship between SDOH domains and advanced cervical cancer stage, defined as stage III/IV based on the International Federation of Gynecology and Obstetrics (FIGO) cervical cancer staging system, at presentation to the Cancer Diseases Hospital (CDH) in Lusaka, the only oncology hospital in Zambia. Women presenting for care for newly diagnosed cervical cancer at CDH were approached for enrollment in this cross-sectional study. They completed a culturally adapted health-related social needs survey, the Accountable Health Communities (AHC) Health-Related Social Needs (HRSN) Screening Tool.^1^ The long-term goal of our study was to understand the relationship of multi-level SDOH domains with advanced-stage cervical cancer at diagnosis.

## METHODS

We conducted an observational, cross-sectional study using a survey adapted from the AHC HRSN Screening Tool developed by the US Centers for Medicare & Medicaid Services.^13–16^ The survey assessed SDOH domains, including the core domains of housing instability, food insecurity, transportation problems, and utility assistance needs, but omitted the interpersonal safety questions to tailor the survey to Zambia’s cultural and ethical context. Questions in the supplemental domain of the HRSN screening tool were also included, such as questions on financial strain, employment, education, physical activity, family and community support, health behaviors or substance use, health literacy and knowledge about cervical cancer, and disabilities. The survey was translated into the 4 most common Zambian languages (Tonga, Nyanga, Lozi, and Bemba).

Patients 18 or older with newly diagnosed cervical cancer who presented for treatment at CDH between June 2022 and March 2025 were approached for participation. After obtaining consent, data were collected through an in-person interview. Each interview lasted approximately 25–35 minutes. The survey was administered in person by a research coordinator and in the patient’s preferred language. A research electronic data capture project (REDCap) database was designed for patient-reported data registration.^17,18^ The study was approved by the University of Zambia’s Biomedical Ethics Research Committee and The University of Texas MD Anderson Cancer Center’s Institutional Review Board.

### Statistical Methods

The primary endpoint of the study was cancer stage at presentation, defined as cancer of the cervix uteri of 2018 FIGO stage III/IV.^19–21^ Descriptive statistics were used to summarize the characteristics of the study population overall and by cancer stage at presentation. Chi-square or Fisher exact tests were performed as appropriate for categorical variables, and Wilcoxon rank-sum tests for continuous variables. A *p* < 0.05 was considered statistically significant.

Logistic regression models examined the associations between advanced cancer stage and SDOH domains. Sociodemographic variables were also explored in the logistic regression models. For the adjusted logistic regression model, we included variables from the unadjusted logistic regression models with an alpha level less than 0.10. Any variable with missing values greater than or equal to 25% were excluded for the adjusted model. All analyses were conducted using Stata/MP 18.0 for Windows (College Station, Texas).

## RESULTS

Between June 2022 and March 2025, 259 patients completed the survey, and 47.1% of them had advanced-stage disease (FIGO stage III/IV). The respondents’ median age was 50 years (range: 29–78), and most patients (79.1%) had completed primary or secondary education (analogous to elementary school or high school in the U.S.), 13.6% had no education, and only 7.4% had completed tertiary education, as reported in Table 1. More than half (55.4%) lived in urban areas (in a town). Patients traveled to CDH from all provinces of Zambia, but mainly were from Lusaka (29.3%), the Copperbelt (17.8%), and the Southern (16.2%), Eastern (13.5%), and Central (11.2%) provinces. The remaining five Zambian provinces each contributed less than 3% of the participating patients. Of the 259 patients surveyed, 143 (55.2%) were women living with human immunodeficiency virus (WLHIV), only 38.8% had prior cervical cancer screening, and 34.4% reported having prior knowledge about their cervical cancer risks and symptoms. There were no significant differences in sociodemographic factors and HIV status between patients with advanced and early FIGO stages. However, some marginally significant differences were found in education and residency. The percentage of early-stage cancer patients with tertiary education was higher than that among patients with less education (10.3% vs. 4.1%; p = 0.057), and a greater proportion of early-stage patients lived in urban areas than in rural areas (61.0% vs. 41.2%; p = 0.056). Patients diagnosed with early-stage disease were significantly more knowledgeable about their cervical cancer risks and symptoms than were advanced-stage patients (41.6% vs. 26.2%, p = 0.009). Among the SDOH domains, early-stage disease patients reported cooking outside or in a building separate from the house significantly less frequently than advanced-stage patients (61.8% vs. 74.6%, p = 0.028), as reported in Table 2.

Our unadjusted models indicated that the patients with no knowledge of cervical cancer symptoms and risks had higher odds of advanced-stage cervical cancer at presentation (OR = 2.00, 95% CI: 1.18–3.40, p = 0.010). Patients who reported cooking outside or in a building separate from the house were 82.0% more likely to have advanced-stage cancer than those who reported cooking in their house (p = 0.029). In the final adjusted model, after adjusting by education, residence, the place the participants cooked, and place where they stayed when they received treatment, the only variable associated with higher odds of advanced-stage cervical cancer at presentation (OR = 1.87, 95% CI: 1.09–3.23, p = 0.023) was knowledge of cervical cancer symptoms and risks. Indicators of stable housing, such as cooking inside the home, was significantly associated with advanced-stage cervical cancer in the unadjusted model but not in the adjusted model (Table 3).

## DISCUSSION

In this cross-sectional study of women with cervical cancer presenting to the Cancer Diseases Hospital in Lusaka, Zambia, we examined the relationship between SDOH and cancer stage at diagnosis. Nearly half of the participants were diagnosed with advanced-stage disease (FIGO stage III/IV). More than 60% of patients reported lacking prior knowledge about cervical cancer risks and symptoms, and this was the only factor significantly associated with advanced-stage presentation in the adjusted model. These findings underscore the critical role of awareness and health literacy in influencing timely care-seeking behavior.

Our results are consistent with those of Lombe et al. (2023), who applied the Three Delays Framework to cancer care in sub-Saharan Africa. Their systematic review identified that delays in seeking, reaching, and receiving quality care are often driven by interconnected barriers, with lack of awareness and low health literacy being dominant contributors to delayed care. Of note, our study primarily captured SDOH factors related to seeking and reaching treatment. Although our survey did not assess system-level barriers, our findings suggest that even women with higher education or urban residence may experience late-stage diagnosis due to uniformly low screening rates and limited access to early detection services. Notably, only 39% of participants reported having undergone cervical cancer screening prior to diagnosis.

In our cohort, none of the standard SDOH domains—including education level, housing stability, transportation access, and rurality—were significantly associated with advanced-stage diagnosis in adjusted models. The strong association between lack of knowledge and advanced-stage disease suggests that informational and behavioral factors may play a more immediate role than structural SDOH in this context. Our results may also be explained by Lombe et al.’s observation that low health literacy, fear, and reliance on traditional medicine are key contributors to delays in seeking care.^22^

Our findings on cervical cancer awareness align with those of previous studies conducted in Zambia and other LMICs. Although cervical cancer screening is freely available in Zambia, coverage remains low. For example, data from the 2021 Zambia Population-Based HIV Impact Assessment survey showed that only 22.2% of women aged 35–49 had undergone cervical cancer screening.^23^ Similarly to our study, Nyambe et al. (2019) reported that only 36.8% of participants (including women and men) were aware of cervical cancer symptoms, and just 38.8% of women in our cohort had undergone screening.^24^ Additionally, lack of awareness has been cited as the primary reason for not undergoing screening.^25,26^ Importantly, while all women are at risk of cervical cancer, women living with HIV (WLHIV) face a significantly higher risk—approximately six times greater than that of HIV-negative women.^27,28^ A study by Mukosha et al. found that HIV status impacts cervical cancer awareness and screening rates; among WLHIV in Lusaka, 62.4% were aware of cervical cancer screening, and 36.5% had been screened, suggesting that WLHIV may be more informed and proactive about screening.^29^ Although WLWH in our cohort demonstrated higher levels of knowledge, this did not translate into earlier presentation; they remained equally likely to be diagnosed at a late stage.

The strengths of our study include its cross-sectional design, the use of a validated survey instrument to assess multiple SDOH domains (e.g., education, transportation, housing, and utility needs), translation of the survey into multiple local languages, and engagement of local staff at CDH, the only cancer hospital in Zambia. Limitations include the relatively small cohort size, which may have limited our power to detect moderate effects in multivariable analyses. Additionally, our sample only included patients who presented to CDH with a cervical cancer diagnosis, which may not be representative of all women living with cervical cancer in Zambia.^23^ Selection bias may have been introduced, as enrollment was conducted during clinical visits and limited to women who spoke one of the four most common languages in Zambia. Finally, recall and reporting bias may have affected survey responses.

## CONCLUSION

Lack of knowledge about the risk and symptoms of cervical cancer was significantly associated with advanced FIGO stage (III/IV) at presentation of cervical cancer among patients in Zambia even after adjusting for other SDOH domains. As described in Lombe et al., future studies are needed to evaluate the interaction between system-level barriers—such as misdiagnosis, poor referral coordination, and limited infrastructure and individual level determinants. Furthermore, additional studies are needed to identify population-based educational interventions as well as interventions focused on increasing screening rates in the general population to improve cervical cancer outcomes in Zambia.

## Figures and Tables

**Table 1:** Characteristics of participants by FIGO cervical cancer stage at diagnosis, early (I/II) vs. advanced (III/IV).

Characteristic	All stages		Early stage		Advanced stage		p-value*
	N = 259		n = 137 (52.9%)		n = 122 (47.1%)		
**Age**, median (range), y	50 (29–78)		51 (29–76)		50 (29–78)		0.473
**Age**, n (%)							0.702
<50	122	47.1	63	46.0	59	48.4	
≥50	137	52.9	74	54.0	63	51.6	
**Marital status,** n (%)							0.808
Single, separated, divorced, or widowed	102	39.4	53	38.7	49	40.2	
Married	157	60.6	84	61.3	73	59.8	
**Education completed,** n (%)							0.057
None/Primary/Secondary	239/258	92.6	122/136	89.7	117	95.9	
Tertiary	19/258	7.4	14/136	10.3	5	4.1	
**Residence,** n (%)							0.056
Urban area (Town)	143/258	55.4	83/136	61.0	60	49.2	
Rural area (Village, Farm)	115/258	44.6	53/136	39.0	62	50.8	
**Province,** n (%)							0.709
Central	29	11.2	13	9.5	16	13.1	
Copperbelt	46	17.8	28	20.4	18	14.8	
Eastern	35	13.5	20	14.6	15	12.3	
Lusaka	76	29.3	41	29.9	35	28.7	
Southern	42	16.2	20	14.6	22	18.0	
Other	31	12.0	15	11.0	16	13.1	
**HIV status,** n (%)							0.555
Not living with HIV	116	44.8	59	43.1	57	46.7	
Living with HIV	143	55.2	78	56.9	65	53.3	
**Prior cervical cancer screening,** n (%)							0.180
Yes	99/255	38.8	58/136	42.6	41/119	34.5	
No	156/255	61.2	78/136	57.4	78/119	65.5	
**Patient’s knowledge about risks and symptoms,** n (%)							0.009
Yes	89	34.4	57	41.6	32	26.2	
No	170	65.6	80	58.4	90	73.8	

FIGO = International Federation of Gynecology and Obstetrics,

* p-values are based on the Wilcoxon rank-sum test for continuous variables and chi-squared test or Fisher exact test for categorical variables. *p* < 0.05 was considered statistically significant.

**Table 2:** Social Determinant of Health by FIGO cervical cancer stage at diagnosis, early (I/II) vs. advanced (III/IV).

Characteristic	All stages		Early stage		Advanced stage		p-value*
	N = 259		n = 137 (52.9%)		n = 122 (47.1%)		
**Living situation**							
Do you own your home? n (%)							0.156
Yes (Purchased, Built)	74	28.6	34	24.8	40	32.8	
No	185	71.4	103	75.2	82	67.2	
House Type, n (%)							0.154
Commercial building/Conventional Flat	150	57.9	85	62.0	65	53.3	
Traditional (mud/thatch)	109	42.1	52	38.0	57	46.7	
What is the main source of energy used for cooking?							0.168
Electricity	45	17.4	28	20.4	17	13.9	
Solar power/Gas/Charcoal/Wood	214	82.6	109	79.6	105	86.1	
Where is the cooking usually done?							0.028
In the house	83/258	32.2	52	38.2	31	25.4	
In a separate building/Outside	175/258	67.8	84	61.8	91	74.6	
From the housing you live, do you have problems with any of the following? Pests such as bugs, ants, mice, lead paints, water leak							0.172
Yes	152	58.7	75	54.7	77	63.1	
No	107	41.3	62	45.3	45	36.9	
**Food**							
Within the past 12 months, have you at some points worried of not having food in the house and not having money to buy more?							0.859
Yes	65	25.1	35	25.5	30	24.6	
No	194	74.9	102	74.5	92	75.4	
**Transportation**							
Where do you stay when you are receiving treatment for your cancer?							0.099
Relatives place in Lusaka	145/256	56.6	83	61.5	62	51.2	
Admitted to the Hospital (CDH)	111/256	43.4	52	38.5	59	48.8	
How do you get to CDH for your appointments or treatment?							0.519
Bus	235/257	91.4	122	90.4	113	92.6	
Other	22/257	8.6	13	9.6	9	7.4	
From the time you were diagnosed with cervical cancer, have you had any challenges with transport to access your hospital medical appointment?							0.225
Yes	209/257	81.3	106	78.5	103	84.4	
No	48/257	18.7	29	21.5	19	15.6	
**Financial strain and income**							
Do you have any source of income?							0.672
Yes	190	73.4	99	72.3	91	74.6	
No	69	26.6	38	27.7	31	25.4	
Has being a cervix cancer patient affected your business or employment?							0.975
Yes	166/185	89.7	88	89.8	78	89.7	
No	19/185	10.3	10	10.2	9	10.3	
Difficulty paying necessities							0.724
Yes	225/257	87.5	120	88.2	105	86.8	
No	32/257	12.5	16	11.8	16	13.2	

FIGO = International Federation of Gynecology and Obstetrics,

* p-values are based on the Wilcoxon rank-sum test for continuous variables and chi-squared test or Fisher exact test for categorical variables. *p* < 0.05 was considered statistically significant.

**Table 3:** Associations of advanced FIGO stagea with social determinants of health and sociodemographic characteristics

	Unadjusted models^b^ (p < 0.1)				Adjusted full model^c^			
	n	OR	95% CI	p-value	n	OR	95% CI	p-value
**Education completed**								
None/Primary/Secondary	239	Ref			235	Ref		
Tertiary	19	0.37	0.13–1.07	0.066	18	0.62	0.20–1.95	0.417
**Residence**								
Urban area (Town)	143				139	Ref		
Rural area (Village, Farm)	115	1.62	0.99–2.65	0.057	114	1.18	0.66–2.10	0.586
**Patient’s knowledge about risks and symptoms**								
Yes	89	Ref			88	Ref		
No	170	2.00	1.18–3.40	0.010	165	1.79	1.03–3.11	0.040
**Cooking place**								
In the house	83	Ref			80	Ref		
Outside or separate building	175	1.82	1.06–3.10	0.029	173	1.56	0.85–2.87	0.149
**Where do you stay when you are receiving treatment for your cancer?**								
Relatives place in Lusaka	145	Ref			142	Ref		
Admitted to the Hospital (CDH)	111	1.52	0.92–2.50	0.099	111	1.18	0.69–1.99	0.550

FIGO = International Federation of Gynecology and Obstetrics, OR = odds ratio, CI = confidence interval, Ref = reference group.

a Advanced stage: FIGO stage III/IV.

b Unadjusted models: univariable logistic regression models including variables with a p-value ≤ 0.10.

c Adjusted model: final logistic regression model.

## Data Availability

CDH being the only radiation oncology center in Zambia increases the risk of re-identification of patients. All relevant summary statistics are provided in the results section, and the survey is enclosed as part of the supplemental methods to help clarify the data characteristics. Further data may be shared upon reasonable request.

## Abbreviations

AHC: Accountable Health Communities
CDH: Cancer Diseases Hospital
HRSN: Health-Related Social Needs
FIGO: International Federation of Gynecology and Obstetrics
LMICs: low- and middle-income countries
SDOH: social determinants of health
WLHIV: women living with human immunodeficiency virus

## References

[1] Social determinants of health. Accessed July 13, 2025. https://www.who.int/health-topics/social-determinants-of-health#tab=tab_1

[2] Askari A, Nachiappan S, Currie A, The relationship between ethnicity, social deprivation and late presentation of colorectal cancer. Cancer Epidemiol. 2017;47:88–93. doi:10.1016/j.canep.2017.01.007 28167416

[3] Daniell JR, Dolja-Gore X, McDowell L, The impact of travel distance to treatment centre on oral tongue squamous cell carcinoma survival and recurrence. Int J Oral Maxillofac Surg. 2022;51(7):854–861. doi:10.1016/j.ijom.2021.08.026 34551874

[4] Han L, Sullivan R, Tree A, The impact of transportation mode, socioeconomic deprivation and rurality on travel times to radiotherapy and surgical services for patients with prostate cancer: A national population-based evaluation. Radiotherapy and Oncology. 2024;192. doi:10.1016/j.radonc.2024.110092 38219910

[5] Zhang X, Zeng Q, Cai W, Ruan W. Trends of cervical cancer at global, regional, and national level: data from the Global Burden of Disease study 2019. BMC Public Health. 2021;21(1). doi:10.1186/S12889-021-10907-5, PMC8114503 33975583

[6] de Oliveira NPD, de Camargo Cancela M, Martins LFL, de Souza DLB. A multilevel assessment of the social determinants associated with the late stage diagnosis of breast cancer. Sci Rep. 2021;11(1):1–9. doi:10.1038/S41598-021-82047-0;SUBJMETA=1347,2195,2324,631,67;KWRD=BREAST+CANCER,CANCER,CANCER+EPIDEMIOLOGY,CANCER+PREVENTION 33526801 PMC7851160

[7] Ssemata AS, Muhumuza R, Seeley J, Moving forward through consensus: a national Delphi approach to determine the top research priorities in prostate cancer in Uganda. BMJ Open. 2023;13(11):e075739. doi:10.1136/BMJOPEN-2023-075739 PMC10689405 38035740

[8] Tekalign T, Teshome M. Prevalence and determinants of late-stage presentation among cervical cancer patients, a systematic review and meta-analysis. PLoS One. 2022;17(4 April). doi:10.1371/JOURNAL.PONE.0267571, PMC9045598 35476851

[9] Cervical cancer | WHO | Regional Office for Africa. Accessed November 12, 2025. https://www.afro.who.int/health-topics/cervical-cancer

[10] Haruyama R, Nyahoda M, Kapambwe S, Sugiura Y, Yokobori Y. Underreported Breast and Cervical Cancer Deaths Among Brought-In-Dead Cases in Zambia. JCO Glob Oncol. 2021;7(7):1209–1211. doi:10.1200/GO.21.00176, 34314228 PMC8457853

[11] Kalima M, Lishimpi K, Meza JL, Observed and Expected Incidence of Cervical Cancer in Lusaka and the Southern and Western Provinces of Zambia, 2007 – 2012. Int J Gynecol Cancer. 2015;25(1):98. doi:10.1097/IGC.0000000000000325 25423318 PMC4272632

[12] Mumba JM, Kasonka L, Owiti OB, Cervical cancer diagnosis and treatment delays in the developing world: Evidence from a hospital-based study in Zambia. Gynecol Oncol Rep. 2021;37. doi:10.1016/j.gore.2021.100784 PMC8165546 34095422

[13] Billioux A, Verlander K, Anthony S, Alley D. Standardized Screening for Health-Related Social Needs in Clinical Settings The Accountable Health Communities Screening Tool. Published online 2017.

[14] Alley DE, Asomugha CN, Conway PH, Sanghavi DM. Accountable Health Communities — Addressing Social Needs through Medicare and Medicaid. New England Journal of Medicine. 2016;374(1):8–11. doi:10.1056/NEJMP1512532 26731305

[15] ZAMBIA 2010 CENSUS OF POPULATION AND HOUSING Report on Characteristics of Households and Housing. Published online 2013. Accessed July 13, 2025. www.zamstats.gov.zm

[16] Nsemukila BG. Summary Report for the 2000 Census of Population and Housing Preface The 2000 Census of Population and Housing was undertaken from 16. Published online 2003. Accessed July 13, 2025. www.zamstats.gov.zm

[17] Harris PA, Taylor R, Minor BL, The REDCap consortium: Building an international community of software platform partners. J Biomed Inform. 2019;95. doi:10.1016/j.jbi.2019.103208 PMC7254481 31078660

[18] Harris PA, Taylor R, Thielke R, Payne J, Gonzalez N, Conde JG. Research electronic data capture (REDCap)-A metadata-driven methodology and workflow process for providing translational research informatics support. J Biomed Inform. 2009;42(2):377–381. doi:10.1016/j.jbi.2008.08.010 18929686 PMC2700030

[19] Bhatla N, Aoki D, Sharma DN, Sankaranarayanan R. Cancer of the cervix uteri. International Journal of Gynecology and Obstetrics. 2018;143:22–36. doi:10.1002/IJGO.12611, 30306584

[20] Bhatla N, Aoki D, Sharma DN, Sankaranarayanan R. Cancer of the cervix uteri: 2021 update. International Journal of Gynecology and Obstetrics. 2021;155(S1):28–44. doi:10.1002/IJGO.13865, 34669203 PMC9298213

[21] Correction to Cancer of the cervix uteri(Int J Gynecol Obstet, (2018), 143, (22–36), 10.1002/ijgo.12611). International Journal of Gynecology and Obstetrics. 2024;164(3):1229–1230. doi:10.1002/IJGO.15395;PAGE:STRING:ARTICLE/CHAPTER 38284793

[22] Lombe DC, Mwamba M, Msadabwe S, Delays in seeking, reaching and access to quality cancer care in sub-Saharan Africa: a systematic review. BMJ Open. 2023;13(4). doi:10.1136/BMJOPEN-2022-067715 PMC10106057 37055211

[23] Lubeya MK, Sinyani A, Mukosha M, Self-Reported Cervical Cancer Screening Uptake Among Women of Reproductive Age in Zambia: Evidence from the 2021 Zambia Population-Based HIV Impact Assessment (ZAMPHIA) Survey. Cancer Control. 2024;31:10732748241307360. doi:10.1177/10732748241307361 PMC11664511 39710618

[24] Nyambe A, Kampen JK, Baboo SK, Van Hal G. Knowledge, attitudes and practices of cervical cancer prevention among Zambian women and men. BMC Public Health. 2019;19(1). doi:10.1186/s12889-019-6874-2 PMC6500583 31054569

[25] Evaluation of cervical cancer screening program at a rural community of South Africa - PubMed. Accessed September 30, 2025. https://pubmed.ncbi.nlm.nih.gov/19024420/19024420

[26] Abiodun OA, Olu-Abiodun OO, Sotunsa JO, Oluwole FA. Impact of health education intervention on knowledge and perception of cervical cancer and cervical screening uptake among adult women in rural communities in Nigeria. BMC Public Health. 2014;14(1):1–9. doi:10.1186/1471-2458-14-814/TABLES/5 25103189 PMC4133628

[27] Stelzle D, Tanaka LF, Lee KK, Estimates of the global burden of cervical cancer associated with HIV. Lancet Glob Health. 2021;9(2):e161–e169. doi:10.1016/S2214-109X(20)30459-9 33212031 PMC7815633

[28] Grulich AE, van Leeuwen MT, Falster MO, Vajdic CM. Incidence of cancers in people with HIV/AIDS compared with immunosuppressed transplant recipients: a meta-analysis. Lancet. 2007;370(9581):59–67. doi:10.1016/S0140-6736(07)61050-2 17617273

[29] Mukosha M, Muyunda D, Mudenda S, Knowledge, attitude and practice towards cervical cancer screening among women living with human immunodeficiency virus: Implication for prevention strategy uptake. Nurs Open. 2023;10(4):2132–2141. doi:10.1002/nop2.1460 36352500 PMC10006627

